# Supplementary material for: Impact of Frailty on the Relationship between Blood Pressure and Cardiovascular Diseases and Mortality in Young-Old Adults
Source: J Pers Med. 2022 Mar 8;12(3):418. doi: 10.3390/jpm12030418 (PMC8949292; doi:10.3390/jpm12030418)
Supplement: Supplementary file 1 [file jpm-12-00418-s001.zip › jpm-1609152-supplementary.pdf]

Table S1. Observational studies

Study	Participant age	Study Size	Outcome	Blood pressure (mm Hg)			Frailty measure (threshold or categories)	Confounders	Main findings
				Categorical		Continuous			
				Systolic	Diastolic				
**Peralta (1)**	>65	3,547	Incident MI; cardiac arrest; stroke; cardiovascular death; all-cause mortality	<120 vs.<br>≥120 & <150<br>≥150	<65 vs.<br>≥65 & <80<br>≥80	10 mmHg difference	ADL; gait speed (fast, ≥1.0 m/s; medium, 0.60–0.99 m/s; slow <0.60 m/s)	Age, sex, race, education, smoking, physical activity, BMI, cholesterol, Cystatin C, antihypertensive medications, and SBP or DBP respectively	The association of BP with CVD or death varied by limitation in performing ADL but not by gait speed.
**Ravindrarajah (2)**	>80	144,403	All-cause mortality	120–139 vs.<br><110<br>110–119<br>140–159<br>≥160			eFI (fit, mild, moderate, severe)	Age, sex, DBP, comorbidity, total cholesterol, and smoking status	SBP <120 mm Hg was associated with greater risk of mortality in both men and women when compared with SBP of 120 to 139 mm Hg. Mortality was higher in more frail participants and the association of SBP <120 mm Hg with mortality was consistently observed at each level of frailty.
**Hospers (3)**	≥65	1,466	All-cause mortality	≤120 vs.<br>>120 & ≤140<br>>140	<70 vs.<br>≥70 & <90<br>≥90		Gait speed (0.8 m/s)	Age, sex, educational level, BMI, smoking, alcohol consumption, total cholesterol, CVD, diabetes and use of antihypertensive drugs	DBP ≤70 mm Hg compared to DBP 71–90mm Hg was associated with an increased all-cause mortality risk. The association was particularly strong in participants with lower levels of both physical and cognitive functioning.
**Masoli (4)**	≥75	415,980	All-cause mortality; incident stroke; MI; cardiac revascularization procedure; heart failure.	130–139 vs.<br><120<br>120–129<br>140–149<br>150–159<br>160–169<br>170–179<br>≥180			eFI	Age, sex, and index of multiple deprivation (IMD)	SBP <130 mm Hg and DBP <80 mm Hg were associated with increased mortality compared to SBP 130–139 mmHg and DBP 80–90 mm Hg in ages above 75 years across all frailty categories. Hypertension was not associated with increased mortality at ages above 85 or 75–84 with moderate/severe frailty.

**Odden (5)**	60–101	1,562	All-cause mortality	<160 vs. ≥160		10 mmHg difference	Self-reported walking speed (slow, medium, fast)	Age, sex, cognitive function, creatinine, interleukin-6, DM medication use, history of MI, stroke, HF, cancer, fracture, and hospitalization	Higher SBP was associated with a 30% elevated risk of all-cause mortality in the fast walkers (p = .004). There was no relationship between higher SBP and mortality in participants who reported slow or medium speed walking.
**Odden (6)**	≥65	2,340	All-cause mortality	<140 vs. ≥140	<90 vs. ≥90	10 mmHg difference	Walking speed (0.8m/s)	Age, sex, CCF, CHD, cholesterol, education, race, smoking, and stroke	The association of BP and mortality varied by walking speed. In fast walkers (non-frail), SBP ≥140 mm Hg was associated with a greater risk of mortality compared with SBP <140 mm Hg (HR 1.35; 95% CI, 1.03–1.77). In slow walkers (frail) neither elevated SBP nor DBP (≥90 mm Hg) were associated with mortality. In the very frail, elevated BP was strongly and independently associated with a lower risk of death.
**Wu (7)**	≥65	7,492	All-cause mortality	<150 vs ≥150	<90 vs. ≥90	10 mmHg difference	grip strength (16 kg for female; 26 kg for male); gait speed (≥0.60 m/s for female; ≥0.52 m/s for male); combination of grip strength and gait speed	Age, sex, BMI, cancer, cardiac disease, DM, education; ethnicity, smoking, stroke, HbA1c, CRP, cystatin C, and antihypertensive medication	Grip strength modified the association of BP with death. Among elders with normal grip strength (≥16 kg for female; ≥26 kg for male), elevated SBP (≥150 mm Hg) and DBP (≥90 mm Hg) were associated with increased mortality risk, whereas there were no significant relationships among those with weak grip strength.
**Gutiérrez-Misis (8)**	⩾65	814	All-cause mortality	<120 vs. 120–140 ≥140	80 vs. 80–90 ≥90		walking speed (≥0.8 m/s)	Age, sex, BMI, total cholesterol, depression, cognitive function, stroke, and HF	Among the slow walkers, there was a decreased risk of mortality for SBP 120–139 mm Hg (HR: 0.39, 95% CI: 0.19–0.77) and SBP ⩾140 mm Hg (HR 0.39, 95%CI: 0.21–0.71), compared with SBP <120 mm Hg.

ADL, activities of daily living; BMI, body mass index; BP, blood pressure; CCF, congestive heart failure; CRP, C-reactive protein; CVD, cardiovascular disease; DBP, diastolic blood presure; DM, diabetes mellitus; eFI, electronic frailty index; HbA1c, glycosylated hemoglobin; HF, heart failure; MI, myocardial infarction; SBP, systolic blood pressure.

Table S2. Incidence and adjusted hazard ratios for myocardial infarction, stroke and mortality by blood pressure (BP)

BP, mmHg		N	Myocardial Infarction				Stroke				Mortality			
			Cases	Duration[a]	IR[b]	HR[c] (95% CI)	Cases	Duration[a]	IR[b]	HR[c] (95% CI)	Cases	Duration[a]	IR[b]	HR[c] (95% CI)
Systolic	<100	10,521	195	69,795	279	1.09 (0.90–1.32)	306	69,403	441	0.93 (0.83–1.04)	624	70,279	888	1.17 (1.08–1.28)*
	100–109	40,661	719	272,487	264	1.02 (0.94–1.11)	1,192	271,188	440	0.92 (0.86–0.98)*	1,874	274,501	683	0.96 (0.91–1.01)
	110–119	126,757	2,257	856,493	264	1.00 (Ref)	4,155	851,452	488	1.00 (Ref)	5,971	863,089	692	1.00 (Ref)
	120–129	167,579	3,080	1,127,316	273	1.02 (0.97–1.08)	5,680	1,119,231	507	1.02 (0.98–1.06)	7,376	1,135,787	649	0.97 (0.93–1.00)
	130–139	205,299	4,066	1,390,456	292	1.07 (1.02–1.13)*	7,909	1,378,569	574	1.13 (1.08–1.17)*	9,647	1,401,771	688	1.02 (0.99–1.06)
	140–149	83,189	1,743	560,730	311	1.10 (1.03–1.17)*	3,607	555,003	650	1.23 (1.17–1.28)*	3,981	565,755	704	1.05 (1.01–1.10)*
	150–159	45,461	1,025	309,085	332	1.15 (1.06–1.23)*	2,265	305,048	743	1.37 (1.30–1.44)*	2,544	311,990	815	1.19 (1.13–1.25)*
	≥160	29,497	742	202,213	367	1.23 (1.13–1.34)*	1,822	198,500	918	1.64 (1.55–1.73)*	1,979	204,269	969	1.37 (1.31–1.45)*
Diastolic	<60	10,735	215	71,088	302	1.12 (0.98–1.29)	324	70,828	457	0.92 (0.80–1.01)	527	71,693	735	0.99 (0.91–1.08)
	60–69	94,531	1,755	632,762	277	1.03 (0.98–1.09)	3,076	628,931	489	0.97 (0.93–1.01)	4,333	637,667	680	0.98 (0.95–1.02)
	70–79	242,029	4,440	1,631,322	272	1.00 (Ref)	8,296	1,619,927	512	1.00 (Ref)	11,083	1,643,723	674	1.00 (Ref)
	80–89	261,471	5,179	1,771,494	292	1.05 (1.01–1.09)*	10,272	1,755,628	585	1.11 (1.08–1.15)*	12,531	1,786,039	702	1.05 (1.02–1.07)*
	90–99	77,474	1,707	526,351	324	1.13 (1.07–1.19)*	3,642	520,105	700	1.28 (1.23–1.33)*	4,047	531,243	762	1.14 (1.10–1.18)*
	100–109	19,310	453	132,192	343	1.17 (1.06–1.29)*	1,088	130,178	836	1.49 (1.40–1.59)*	1,226	133,485	918	1.35 (1.27–1.43)*
	≥110	3,414	78	23,368	334	1.10 (0.88–1.38)	238	22,796	1,044	1.79 (1.58–2.04)*	249	23,591	1,056	1.46 (1.28–1.65)*

Abbreviations: aHR, adjusted hazard ratio; IR, incidence rate

[a]Person-years.

[b]Per 100,000 person-years.

[c]Adjusted for sex, BMI, smoking, hemoglobin level, alcohol drinking, regular exercise, income, antihypertensive medication use, diabetes, chronic kidney disease and chronic pulmonary obstructive disease.

*Statistically significant value

Table S3. Incidence and adjusted hazard ratios for myocardial infarction, stroke and total mortality by systolic blood pressure (SBP) and Timed Up and Go (TUG)

TUG, sec	SBP, mmHg	N	Myocardial Infarction				Stroke				Total Mortality			
			Cases	Duration[a]	IR[b]	HR[c] (95% CI)	Cases	Duration[a]	IR[b]	HR[c] (95% CI)	Cases	Duration[a]	IR[b]	HR[c] (95% CI)
<10	<100	7,782	143	51,555	277	1.09 (0.92–1.30)	218	51,272	425	0.92 (0.80–1.06)	438	51,915	844	1.17 (1.06–1.29)*
	100–109	30,329	524	202,899	258	1.01 (0.91–1.11)	877	201,860	434	0.94 (0.87–1.01)	1,326	204,299	649	0.95 (0.90–1.01)
	110–119	95,316	1,673	642,284	260	1.00 (Ref)	3,012	638,770	472	1.00 (Ref)	4,284	647,183	662	1.00 (Ref)
	120–129	121,465	2,122	814,111	261	0.99 (0.93–1.05)	3,881	808,782	480	1.00 (0.95–1.05)	5,169	819,959	630	0.98 (0.94–1.02)
	130–139	149,631	2,869	1,008,643	284	1.06 (0.99–1.12)	5,555	1,000,471	555	1.13 (1.08–1.18)*	6,751	1,016,586	664	1.03 (0.99–1.07)
	140–149	59,781	1,222	401,697	304	1.09 (1.01–1.17)*	2,547	397,712	640	1.25 (1.19–1.32)*	2,755	405,217	680	1.06 (1.01–1.11)*
	150–159	32,840	715	222,540	321	1.12 (1.03–1.23)*	1,585	219,710	721	1.37 (1.29–1.46)*	1,776	224,606	791	1.20 (1.13–1.27)*
	≥160	21,056	512	144,064	355	1.21 (1.10–1.34)*	1,241	141,562	877	1.62 (1.51–1.73)*	1,378	145,482	947	1.40 (1.32–1.49)*
10–14	<100	2,389	47	15,932	295	1.16 (0.87–1.56)	74	15,846	467	1.02 (0.81–1.28)	163	16,041	1016	1.43 (1.22–1.67)*
	100–109	9,047	167	60,931	274	1.07 (0.91–1.25)	272	60,714	448	0.97 (0.85–1.09)	471	61,458	766	1.14 (1.04–1.25)*
	110–119	27,676	504	188,549	267	1.02 (0.92–1.12)	1,004	187,173	536	1.13 (1.05–1.22)*	1,466	189,989	772	1.18 (1.11–1.25)*
	120–129	40,479	829	274,722	302	1.13 (1.04–1.23)*	1,542	272,345	566	1.18 (1.11–1.25)*	1,913	276,980	691	1.09 (1.03–1.15)*
	130–139	48,590	1,014	333,089	304	1.11 (1.03–1.20)*	2,045	329,806	620	1.25 (1.18–1.32)*	2,491	335,917	742	1.16 (1.10–1.22)*
	140–149	20,230	429	137,351	312	1.11 (0.99–1.23)*	910	135,796	670	1.30 (1.21–1.41)*	1,024	138,576	739	1.18 (1.10–1.26)*
	150–159	10,963	257	75,164	342	1.19 (1.04–1.36)*	582	74,107	785	1.50 (1.37–1.64)*	647	75,868	853	1.33 (1.22–1.45)*
	≥160	7,255	189	49,929	379	1.28 (1.10–1.48)*	484	48,903	990	1.82 (1.66–2.01)*	517	50,449	1,025	1.54 (1.41–1.69)*
≥15	<100	350	5	2,308	217	0.85 (0.35–2.04)	14	2,285	613	1.32 (0.78–2.23)	23	2,323	990	1.40 (0.93–2.10)
	100–109	1,285	28	8,657	323	1.22 (0.84–1.77)	43	8,614	499	1.05 (0.78–1.42)	77	8,745	880	1.29 (1.03–1.61)*
	110–119	3,765	80	25,661	312	1.16 (0.93–1.45)	139	25,508	545	1.13 (0.95–1.33)	221	25,917	853	1.30 (1.13–1.49)*
	120–129	5,635	129	38,483	335	1.22 (1.02–1.46)*	257	38,104	674	1.37 (1.20–1.55)*	294	38,848	757	1.17 (1.04–1.31)*
	130–139	7,078	183	48,724	376	1.34 (1.15–1.56)*	309	48,291	640	1.27 (1.13–1.43)*	405	49,268	822	1.28 (1.16–1.42)*
	140–149	3,178	92	21,682	424	1.47 (1.19–1.81)*	150	21,495	698	1.33 (1.13–1.57)*	202	21,963	920	1.46 (1.27–1.68)*
	150–159	1,658	53	11,382	466	1.59 (1.21–2.09)*	98	11,231	873	1.64 (1.34–2.00)*	121	11,516	1,051	1.65 (1.37–1.97)*
	≥160	1,186	41	8,220	499	1.66 (1.21–2.26)*	97	8,035	1207	2.18 (1.78–2.67)*	84	8,339	1,007	1.53 (1.23–1.90)*

Abbreviations: aHR, adjusted hazard ratio; IR, incidence rate; sec, seconds

[a]Person-years.

[b]Per 100,000 person-years.

[c]Adjusted for sex, BMI, smoking, hemoglobin level, alcohol drinking, regular exercise, income, antihypertensive medication use, diabetes, chronic kidney disease and chronic pulmonary obstructive disease.

*Statistically significant value

Table S4. Incidence and adjusted hazard ratios for myocardial infarction, stroke and total mortality by diastolic blood pressure (DBP) and Timed Up and Go (TUG)

TUG, sec	DBP, mmHg	N	Myocardial Infarction				Stroke				Total Mortality			
			Cases	Duration[a]	IR[b]	HR[c] (95% CI)	Cases	Duration[a]	IR[b]	HR[c] (95% CI)	Cases	Duration[a]	IR[b]	HR[c] (95% CI)
	<60	7,746	152	51,241	297	1.13 (0.96–1.33)	216	51,086	423	0.87 (0.76–0.99)*	358	51,654	693	0.97 (0.87–1.08)
	60–69	69,919	1,273	467,334	272	1.04 (0.97–1.11)	2,206	464,524	475	0.98 (0.93–1.03)	3,015	470,866	640	0.95 (0.91–0.99)*
	70–79	179,904	3,222	1,209,114	266	1.00 (Ref)	5,914	1,201,232	492	1.00 (Ref)	7,979	1,218,092	655	1.00 (Ref)
<10	80–89	189,213	3,617	1,275,681	284	1.04 (0.99–1.09)	7,159	1,264,988	566	1.12 (1.08–1.16)*	8,760	1,285,826	681	1.04 (1.01–1.08)*
	90–99	55,331	1,152	374,774	307	1.09 (1.02–1.17)*	2,507	370,401	677	1.29 (1.23–1.35)*	2,732	378,102	723	1.11 (1.07–1.16)*
	100–109	13,681	309	93,224	331	1.15 (1.02–1.29)*	750	91,869	816	1.51 (1.40–1.63)*	857	94,125	910	1.36 (1.27–1.50)*
	≥110	2,406	55	16,426	335	1.14 (0.87–1.48)	164	16,039	1,022	1.83 (1.57–2.14)*	176	16,580	1,062	1.54 (1.33–1.79)*
	<60	2,627	56	17,516	320	1.18 (0.91–1.54)	91	17,421	522	1.05 (0.85–1.29)	134	17,678	758	1.06 (0.90–1.25)
	60–69	21,544	422	144,739	292	1.10 (0.99–1.22)	745	143,900	518	1.06 (0.98–1.44)	1,143	145,950	783	1.18 (1.11–1.25)*
	70–79	54,607	1,041	370,979	281	1.04 (0.97–1.12)	2,065	367,911	561	1.13 (0.08–1.19)	2,703	373,867	723	1.12 (1.07–1.17)*
10–14	80–89	62,988	1,308	432,125	303	1.10 (1.03–1.17)*	2,679	427,520	627	1.23 (1.18–1.29)*	3,227	435,770	741	1.15 (1.11–1.20)*
	90–99	19,152	473	130,859	361	1.27 (1.15–1.40)*	977	129,232	756	1.43 (1.34–1.53)*	1,106	132,183	837	1.31 (1.23–1.40)*
	100–109	4,848	116	33,521	346	1.21 (1.01–1.46)*	293	32,930	890	1.66 (1.48–1.87)*	314	33,842	928	1.45 (1.30–1.62)*
	≥110	863	20	5,927	337	1.13 (0.72–1.75)	63	5,776	1,091	1.93 (1.51–2.48)*	65	5,987	1,086	1.50 (1.18–1.92)*
	<60	362	7	2,330	300	1.13 (0.54–2.38)	17	2,320	733	1.50 (0.93–2.41)	35	2,361	1,483	2.12 (1.52–2.96)*
	60–69	3,068	60	20,689	290	1.05 (0.81–1.35)	125	20,508	610	1.21 (1.01–1.44)*	175	20,851	839	1.24 (1.07–1.44)*
	70–79	7,518	177	51,229	346	1.25 (1.07–1.45)*	317	50,784	624	1.23 (1.10–1.37)*	401	51,764	775	1.19 (1.08–1.31)*
≥15	80–89	9,270	254	63,688	399	1.42 (1.25–1.61)*	434	63,120	688	1.33 (1.20–1.46)*	544	64,442	844	1.30 (1.89–1.41)*
	90–99	2,991	82	20,718	396	1.36 (1.10–1.70)*	158	20,473	772	1.43 (1.22–1.68)*	209	20,958	997	1.56 (1.36–1.79)*
	100–109	781	28	5,447	514	1.79 (1.24–2.60)*	45	5,379	837	1.57 (1.17–2.10)*	55	5,518	997	1.66 (1.27–2.16)*
	≥110	145	3	1,016	295	0.92 (0.30–2.86)	11	981	1,122	1.87 (1.04–3.39)*	8	1,024	781	1.01 (0.50–2.01)

Abbreviations: aHR, adjusted hazard ratio; IR, incidence rate; sec, seconds

[a]Person–years.

[b]Per 100,000 person–years.

[c]Adjusted for sex, BMI, smoking, hemoglobin level, alcohol drinking, regular exercise, income, antihypertensive medication use, diabetes, chronic kidney disease and chronic pulmonary obstructive disease.

*Statistically significant value

Table S5. Incidence and adjusted hazard ratios for myocardial infaction, stroke and total mortality in different systolic blood pressure (SBP) groups in each Timed Up and Go (TUG) group

TUG, sec	SBP, mmHg	N	Myocardial Infarction				Stroke				Death			
			Cases	Duration[a]	IR[b]	HR[c] (95% CI)	Cases	Duration[a]	IR[b]	aHR[c] (95% CI)	Cases	Duration[a]	IR[b]	aHR[c] (95% CI)
<10	<100	7,782	143	51,555	277	1.10 (0.93–1.31)	218	51,272	425	0.93 (0.81–1.06)	438	51,915	844	1.17 (1.06–1.29)*
	100–109	30,329	524	202,899	258	1.01 (0.92–1.12)	877	201,860	434	0.94 (0.87–1.01)	1,326	204,299	649	0.95 (0.90–1.01)
	110–119	95,316	1,673	642,284	260	1.00 (Ref)	3,012	638,770	472	1.00 (Ref)	4,284	647,183	662	1.00 (Ref)
	120–129	121,465	2,122	814,111	261	0.99 (0.92–1.05)	3,881	808,782	480	1.00 (0.95–1.05)	5,169	819,959	630	0.98 (0.94–1.02)
	130–139	149,631	2,869	1,008,643	284	1.05 (0.99–1.12)	5,555	1,000,471	555	1.13 (1.08–1.18)*	6,751	1,016,586	664	1.03 (0.99–1.07)
	140–149	59,781	1,222	401,697	304	1.09 (1.01–1.17)*	2,547	397,712	640	1.25 (1.19–1.32)*	2,755	405,217	680	1.06 (1.01–1.11)*
	150–159	32,840	715	222,540	321	1.12 (1.02–1.22)*	1,585	219,710	721	1.37 (1.29–1.46)*	1,776	224,606	791	1.20 (1.13–1.27)*
	≥160	21,056	512	144,064	355	1.21 (1.09–1.34)*	1,241	141,562	877	1.62 (1.51–1.73)*	1,378	145,482	947	1.40 (1.32–1.49)*
10–14	<100	2,389	47	15,932	295	1.12 (0.83–1.52)	74	15,846	467	0.89 (0.71–1.13)	163	16,041	1,016	1.20 (1.02–1.42)*
	100–109	9,047	167	60,931	274	1.04 (0.87–1.24)	272	60,714	448	0.85 (0.75–0.98)*	471	61,458	766	0.96 (0.87–1.07)
	110–119	27,676	504	188,549	267	1.00 (Ref)	1,004	187,173	536	1.00 (Ref)	1,466	189,989	772	1.00 (Ref)
	120–129	40,479	829	274,722	302	1.12 (1.00–1.25)*	1,542	272,345	566	1.04 (0.96–1.12)	1,913	276,980	691	0.92 (0.86–0.99)*
	130–139	48,590	1,014	333,089	304	1.09 (0.98–1.22)	2,045	329,806	620	1.10 (1.02–1.19)*	2,491	335,917	742	0.98 (0.92–1.05)
	140–149	20,230	429	137,351	312	1.09 (0.96–1.24)	910	135,796	670	1.15 (1.05–1.26)*	1,024	138,576	739	1.00 (0.92–1.09)
	150–159	10,963	257	75,164	342	1.17 (1.01–1.36)*	582	74,107	785	1.32 (1.19–1.46)*	647	75,868	853	1.13 (1.03–1.24)*
	≥160	7,255	189	49,929	379	1.25 (1.06–1.48)*	484	48,903	990	1.61 (1.44–1.79)*	517	50,449	1,025	1.31 (1.18–1.45)*
≥15	<100	350	5	2,308	217	0.72 (0.29–1.78)	14	2,285	613	1.12 (0.65–1.95)	23	2,323	990	1.07 (0.70–1.65)
	100–109	1,285	28	8,657	323	1.04 (0.68–1.60)	43	8,614	499	0.92 (0.65–1.29)	77	8,745	880	0.98 (0.75–1.27)
	110–119	3,765	80	25,661	312	1.00 (Ref)	139	25,508	545	1.00 (Ref)	221	25,917	853	1.00 (Ref)
	120–129	5,635	129	38,483	335	1.06 (0.80–1.40)	257	38,104	674	1.21 (0.98–1.49)	294	38,848	757	0.89 (0.75–1.06)
	130–139	7,078	183	48,724	376	1.17 (0.90–1.53)	309	48,291	640	1.14 (0.93–1.39)	405	49,268	822	0.98 (0.83–1.16)
	140–149	3,178	92	21,682	424	1.29 (0.95–1.75)	150	21,495	698	1.19 (0.94–1.50)	202	21,963	920	1.11 (0.91–1.34)
	150–159	1,658	53	11,382	466	1.39 (0.98–1.97)	98	11,231	873	1.46 (1.13–1.90)*	121	11,516	1,051	1.26 (1.01–1.58)*
	≥160	1,186	41	8,220	499	1.43 (0.97–2.09)	97	8,035	1,207	1.92 (1.48–2.50)*	84	8,339	1,007	1.16 (0.90–1.50)

Abbreviations: aHR, adjusted hazard ratio; IR, incidence rate; sec, seconds

[a]Person–years.

[b]Per 100,000 person–years.

[c]Adjusted for sex, BMI, smoking, hemoglobin level, alcohol drinking, regular exercise, income, antihypertensive medication use, diabetes, chronic kidney disease and chronic pulmonary obstructive disease.

*Statistically significant value.

Table S6. Incidence and adjusted hazard ratios for myocardial infarction, stroke and total mortality in different diastolic blood pressure (DBP) groups in each Timed Up and Go (TUG) group

TUG, sec	DBP, mmHg	N	Myocardial Infarction				Stroke				Death			
			Cases	Duration[a]	IR[b]	HR[c] (95% CI)	Cases	Duration[a]	IR[b]	HR[c] (95% CI)	Cases	Duration[a]	IR[b]	HR[c] (95% CI)
	<60	7,746	152	51,241	297	1.14 (0.96–1.34)	216	51,086	113	0.87 (0.76–1.00)	358	51,654	113	0.97 (0.87–1.08)
	60–69	69,919	1,273	467,334	272	1.04 (0.97–1.11)	2,206	464,524	104	0.98 (0.94–1.03)	3,015	470,866	104	0.95 (0.91–0.99)*
	70–79	179,904	3,222	1,209,114	266	1.00 (Ref)	5,914	1,201,232	100	1.00 (Ref)	7,979	1,218,092	100	1.00 (Ref)
<10	80–89	189,213	3,617	1,275,681	284	1.04 (0.99–1.09)	7,159	1,264,988	104	1.12 (1.08–1.16)*	8,760	1,285,826	104	1.04 (1.01–1.08)*
	90–99	55,331	1,152	374,774	307	1.09 (1.02–1.17)*	2,507	370,401	109	1.28 (1.22–1.35)*	2,732	378,102	109	1.11 (1.06–1.16)*
	100–109	13,681	309	93,224	331	1.14 (1.02–1.29)*	750	91,869	115	1.50 (1.39–1.62)*	857	94,125	115	1.36 (1.26–1.46)*
	≥110	2,406	55	16,426	335	1.13 (0.87–1.48)	164	16,039	114	1.83 (1.57–2.14)*	176	16,580	114	1.54 (1.32–1.79)*
	<60	2,627	56	17,516	320	1.11 (0.85–1.46)	91	17,421	118	0.93 (0.75–1.14)	134	17,678	118	0.94 (0.79–1.12)
	60–69	21,544	422	144,739	292	1.05 (0.93–1.17)	745	143,900	110	0.94 (0.86–1.02)	1,143	145,950	110	1.05 (0.98–1.13)
	70–79	54,607	1,041	370,979	281	1.00 (Ref)	2,065	367,911	104	1.00 (Ref)	2,703	373,867	104	1.00 (Ref)
10–14	80–89	62,988	1,308	432,125	303	1.06 (0.98–1.15)	2,679	427,520	110	1.09 (1.03–1.15)*	3,227	435,770	110	1.03 (0.98–1.08)
	90–99	19,152	473	130,859	361	1.23 (1.10–1.37)*	977	129,232	127	1.27 (1.17–1.37)*	1,106	132,183	127	1.17 (1.09–1.26)*
	100–109	4,848	116	33,521	346	1.17 (0.96–1.42)*	293	32,930	121	1.47 (1.30–1.66)*	314	33,842	121	1.30 (1.16–1.46)*
	≥110	863	20	5,927	337	1.09 (0.70–1.70)	63	5,776	113	1.70 (1.32–2.19)*	65	5,987	113	1.35 (1.05–1.72)*
	<60	362	7	2,330	300	0.89 (0.42–1.89)	17	2,320	113	1.19 (0.73–1.93)	35	2,361	113	1.76 (1.25–2.50)*
	60–69	3,068	60	20,689	290	0.84 (0.62–1.12)	125	20,508	105	0.96 (0.78–1.19)	175	20,851	105	1.03 (0.86–1.23)
	70–79	7,518	177	51,229	346	1.00 (Ref)	317	50,784	125	1.00 (Ref)	401	51,764	125	1.00 (Ref)
≥15	80–89	9,270	254	63,688	399	1.14 (0.94–1.38)	434	63,120	142	1.09 (0.94–1.26)	544	64,442	142	1.09 (0.96–1.24)
	90–99	2,991	82	20,718	396	1.10 (0.84–1.43)	158	20,473	136	1.17 (0.96–1.42)	209	20,958	136	1.31 (1.11–1.55)*
	100–109	781	28	5,447	514	1.43 (0.96–2.14)	45	5,379	179	1.30 (0.95–1.78)	55	5,518	179	1.40 (1.05–1.86)*
	≥110	145	3	1,016	295	0.74 (0.24–2.31)	11	981	92	1.49 (0.82–2.73)	8	1,024	92	0.84 (0.42–1.70)

Abbreviations: aHR, adjusted hazard ratio; IR, incidence rate

[a] Person–years.

[b] Per 100,000 person–years.

[c] Adjusted for sex, BMI, smoking, hemoglobin level, alcohol drinking, regular exercise, income, antihypertensive medication use, diabetes, chronic kidney disease and chronic pulmonary obstructive disease.

*Statistically significant value.

(A) Myocardial Infarction (SBP x TUG)

TUG, sec	SBP, mm Hg	Hazard Ratio (95% CI) Antihypertensive drug: No	Yes
<10	<100	1.12 (0.91–1.36)	1.15 (0.82–1.61)
	100–109	1.00 (0.89–1.13)	1.08 (0.91–1.27)
	**110–119**	**1.00 (Ref)**	**1.00 (Ref)**
	120–129	1.03 (0.95–1.12)	0.91 (0.82–1.01)
	130–139	1.14 (1.05–1.23)*	0.94 (0.86–1.03)
	140–149	1.23 (1.10–1.37)*	0.95 (0.85–1.05)
	150–159	1.23 (1.07–1.42)*	1.00 (0.89–1.13)
	≥160	1.50 (1.28–1.76)*	1.02 (0.90–1.17)
10–14	<100	1.13 (0.80–1.60)	1.40 (0.82–2.38)
	100–109	1.10 (0.91–1.33)	1.04 (0.78–1.38)
	110–119	1.07 (0.95–1.22)	0.93 (0.79–1.10)
	120–129	1.14 (1.02–1.28)*	1.09 (0.96–1.24)
	130–139	1.14 (1.02–1.27)*	1.04 (0.92–1.16)
	140–149	1.30 (1.11–1.52)*	0.94 (0.81–1.09)
	150–159	1.10 (0.87–1.39)	1.15 (0.98–1.36)
	≥160	1.37 (1.05–1.79)	1.15 (0.96–1.39)
≥15	<100	0.94 (0.35–2.51)	0.69 (0.10–4.94)
	100–109	1.37 (0.88–2.13)	0.99 (0.49–1.98)
	110–119	1.19 (0.89–1.59)	1.10 (0.78–1.56)
	120–129	1.33 (1.05–1.68)*	1.08 (0.82–1.41)
	130–139	1.47 (1.18–1.82)*	1.18 (0.95–1.47)
	140–149	1.91 (1.39–2.63)*	1.18 (0.89–1.56)
	150–159	1.06 (0.59–1.92)	1.70 (1.24–2.32)*
	≥160	1.65 (0.93–2.92)	1.55 (1.07–2.24)*

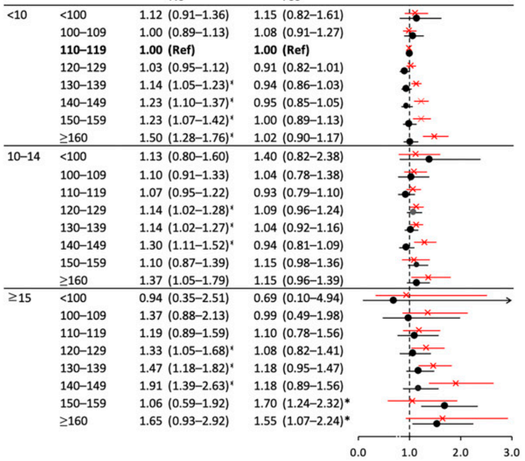

(B) Stroke (SBP x TUG)

TUG, sec	SBP, mm Hg	Hazard Ratio (95% CI) Antihypertensive drug: No	Yes
<10	<100	0.90 (0.76–1.06)	1.12 (0.87–1.44)
	100–109	0.93 (0.84–1.02)	1.00 (0.88–1.14)
	**110–119**	**1.00 (Ref)**	**1.00 (Ref)**
	120–129	1.03 (0.97–1.10)	0.94 (0.87–1.01)
	130–139	1.23 (1.16–1.30)*	0.99 (0.93–1.06)
	140–149	1.44 (1.34–1.56)*	1.07 (0.99–1.16)
	150–159	1.56 (1.42–1.71)*	1.19 (1.10–1.30)*
	≥160	1.95 (1.76–2.17)*	1.37 (1.26–1.50)*
10–14	<100	0.93 (0.70–1.24)	1.37 (0.92–2.04)
	100–109	0.90 (0.77–1.06)	1.14 (0.93–1.39)
	110–119	1.19 (1.09–1.30)*	1.05 (0.93–1.18)
	120–129	1.21 (1.12–1.32)*	1.10 (1.00–1.20)*
	130–139	1.28 (1.19–1.39)*	1.15 (1.06–1.25)*
	140–149	1.51 (1.35–1.69)*	1.12 (1.01–1.24)*
	150–159	1.79 (1.56–2.06)*	1.27 (1.13–1.43)*
	≥160	2.23 (1.90–2.62)*	1.54 (1.36–1.74)*
≥15	<100	0.90 (0.43–1.90)	2.82 (1.34–5.94)*
	100–109	1.19 (0.84–1.70)	0.82 (0.47–1.45)
	110–119	1.10 (0.88–1.38)	1.13 (0.88–1.47)
	120–129	1.40 (1.17–1.66)*	1.29 (1.07–1.55)*
	130–139	1.33 (1.13–1.58)*	1.15 (0.98–1.36)
	140–149	1.44 (1.10–1.89)*	1.19 (0.97–1.47)
	150–159	1.86 (1.33–2.60)*	1.43 (1.11–1.84)*
	≥160	2.14 (1.47–3.11)*	2.04 (1.60–2.60)*

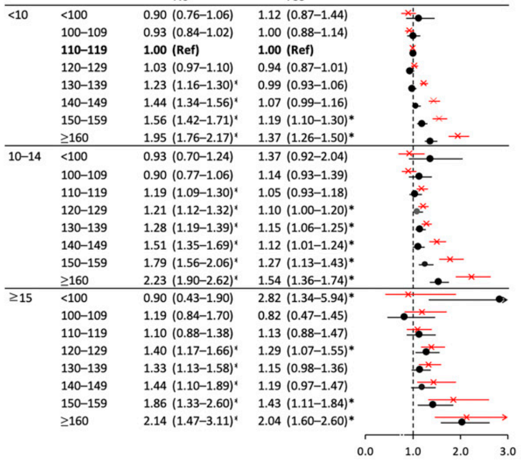

(C) Death (SBP x TUG)

TUG, sec	SBP, mm Hg	Hazard Ratio (95% CI) Antihypertensive drug: No	Yes
<10	<100	1.14 (1.02–1.27)*	1.31 (1.02–1.27)*
	100–109	0.92 (0.86–0.99)*	1.06 (0.86–0.99)*
	**110–119**	**1.00 (Ref)**	**1.00 (Ref)**
	120–129	0.96 (0.91–1.01)	1.01 (0.91–1.01)
	130–139	1.07 (1.02–1.13)*	0.97 (1.02–1.13)*
	140–149	1.11 (1.04–1.19)*	1.01 (1.04–1.19)*
	150–159	1.24 (1.15–1.35)*	1.15 (1.15–1.35)*
	≥160	1.50 (1.37–1.65)*	1.32 (1.37–1.65)*
10–14	<100	1.38 (1.15–1.65)*	1.64 (1.15–1.65)*
	100–109	1.12 (1.01–1.26)*	1.19 (1.01–1.26)*
	110–119	1.20 (1.11–1.28)*	1.15 (1.11–1.28)*
	120–129	1.10 (1.03–1.17)*	1.07 (1.03–1.17)*
	130–139	1.13 (1.06–1.21)*	1.18 (1.06–1.21)*
	140–149	1.32 (1.20–1.46)*	1.06 (1.20–1.46)*
	150–159	1.47 (1.30–1.67)*	1.23 (1.30–1.67)*
	≥160	1.84 (1.59–2.11)*	1.36 (1.59–2.11)*
≥15	<100	1.38 (0.88–2.17)	1.50 (0.88–2.17)
	100–109	1.14 (0.86–1.51)	1.69 (0.86–1.51)
	110–119	1.31 (1.10–1.54)*	1.28 (1.10–1.54)*
	120–129	1.10 (0.94–1.29)	1.25 (0.94–1.29)
	130–139	1.16 (1.00–1.34)*	1.40 (1.00–1.34)*
	140–149	1.63 (1.32–2.02)*	1.32 (1.32–2.02)*
	150–159	1.95 (1.49–2.56)*	1.43 (1.49–2.56)*
	≥160	1.78 (1.26–2.51)*	1.37 (1.26–2.51)*

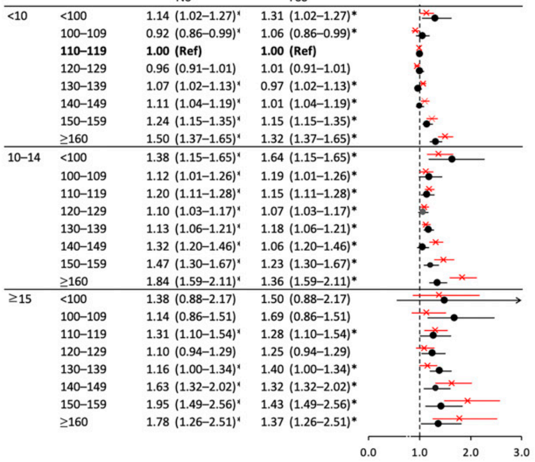

(D) Myocardial Infarction (DBP x TUG)

TUG, sec	DBP, mm Hg	Hazard Ratio (95% CI) Antihypertensive drug: No	Yes
<10	<60	1.19 (0.97–1.45)	1.06 (0.80–1.41)
	60–69	1.04 (0.95–1.13)	1.05 (0.95–1.16)
	**70–79**	**1.00 (Ref)**	**1.00 (Ref)**
	80–89	1.11 (1.04–1.18)*	0.97 (0.90–1.04)
	90–99	1.17 (1.06–1.30)*	1.03 (0.94–1.12)
	100–109	1.18 (0.97–1.44)	1.11 (0.95–1.28)
	≥110	1.13 (0.69–1.85)	1.12 (0.81–1.54)
10–14	<60	1.21 (0.87–1.69)	1.17 (0.76–1.80)
	60–69	1.08 (0.95–1.24)	1.14 (0.97–1.33)
	70–79	1.04 (0.94–1.14)	1.05 (0.95–1.16)
	80–89	1.12 (1.02–1.22)*	1.06 (0.97–1.17)
	90–99	1.39 (1.19–1.61)*	1.18 (1.04–1.34)*
	100–109	1.28 (0.93–1.74)	1.15 (0.92–1.45)
	≥110	1.60 (0.83–3.08)	0.89 (0.49–1.60)
≥15	<60	1.71 (0.77–3.81)	0.38 (0.05–2.70)
	60–69	1.16 (0.84–1.61)	0.91 (0.60–1.38)
	70–79	1.30 (1.06–1.60)*	1.19 (0.95–1.48)
	80–89	1.45 (1.21–1.75)*	1.37 (1.15–1.64)*
	90–99	1.32 (0.91–1.91)	1.37 (1.04–1.80)*
	100–109	1.75 (0.94–3.26)	1.78 (1.12–2.84)*
	≥110	1.34 (0.19–9.54)	0.77 (0.19–3.08)

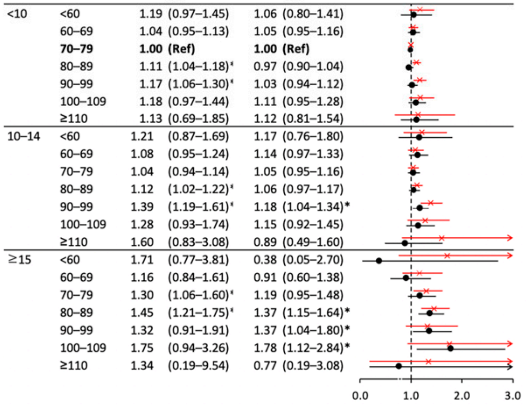

(E) Stroke (DBP x TUG)

TUG, sec	DBP, mm Hg	Hazard Ratio (95% CI) Antihypertensive drug: No	Yes
<10	<60	0.79 (0.66–0.95)	1.04 (0.84–1.28)
	60–69	0.96 (0.90–1.02)	1.04 (0.96–1.12)
	**70–79**	**1.00 (Ref)**	**1.00 (Ref)**
	80–89	1.19 (1.13–1.24)*	1.05 (0.99–1.10)
	90–99	1.36 (1.27–1.46)*	1.22 (1.14–1.30)*
	100–109	1.69 (1.49–1.91)*	1.39 (1.26–1.53)*
	≥110	2.06 (1.57–2.69)*	1.70 (1.41–2.06)*
10–14	<60	1.14 (0.89–1.48)	0.93 (0.65–1.32)
	60–69	1.04 (0.94–1.15)	1.11 (0.99–1.25)
	70–79	1.16 (1.08–1.24)*	1.10 (1.03–1.19)*
	80–89	1.23 (1.15–1.31)*	1.22 (1.14–1.30)*
	90–99	1.61 (1.45–1.78)*	1.31 (1.19–1.43)*
	100–109	2.02 (1.68–2.43)*	1.45 (1.25–1.69)*
	≥110	2.24 (1.49–3.38)*	1.76 (1.29–2.40)*
≥15	<60	1.54 (0.83–2.86)	1.47 (0.70–3.08)
	60–69	1.18 (0.93–1.50)	1.24 (0.96–1.62)
	70–79	1.21 (1.03–1.41)*	1.25 (1.06–1.47)*
	80–89	1.26 (1.09–1.46)*	1.37 (1.20–1.56)*
	90–99	1.64 (1.28–2.10)*	1.30 (1.05–1.59)*
	100–109	2.01 (1.31–3.09)*	1.30 (0.87–1.94)
	≥110	1.44 (0.36–5.78)	1.93 (1.01–3.72)*

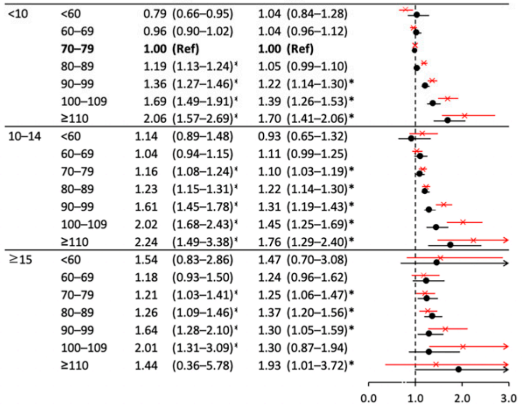

(F) Death (DBP x TUG)

TUG, sec	DBP, mm Hg	Hazard Ratio (95% CI) Antihypertensive drug: No	Yes
<10	<60	0.96 (0.84–1.09)	1.00 (0.83–1.21)
	60–69	0.94 (0.89–0.99)*	0.99 (0.92–1.06)
	**70–79**	**1.00 (Ref)**	**1.00 (Ref)**
	80–89	1.07 (1.03–1.12)*	1.00 (0.96–1.05)
	90–99	1.18 (1.11–1.26)*	1.05 (0.99–1.11)
	100–109	1.34 (1.20–1.49)*	1.35 (1.23–1.49)*
	≥110	1.27 (0.96–1.68)	1.66 (1.39–1.98)*
10–14	<60	1.07 (0.87–1.31)	1.05 (0.78–1.42)
	60–69	1.19 (1.10–1.28)*	1.17 (1.05–1.29)*
	70–79	1.15 (1.08–1.21)*	1.08 (1.01–1.16)*
	80–89	1.16 (1.09–1.22)*	1.14 (1.07–1.21)*
	90–99	1.41 (1.29–1.55)*	1.22 (1.12–1.33)*
	100–109	1.60 (1.34–1.90)*	1.34 (1.16–1.56)*
	≥110	1.84 (1.29–2.64)*	1.29 (0.92–1.79)
≥15	<60	2.15 (1.41–3.26)*	2.11 (1.22–3.63)*
	60–69	1.15 (0.94–1.40)	1.38 (1.10–1.73)*
	70–79	1.18 (1.04–1.35)*	1.19 (1.02–1.39)*
	80–89	1.24 (1.10–1.40)*	1.34 (1.18–1.52)*
	90–99	1.68 (1.36–2.06)*	1.47 (1.22–1.76)*
	100–109	1.47 (0.96–2.25)	1.76 (1.26–2.47)*
	≥110	1.58 (0.51–4.90)	0.80 (0.33–1.93)

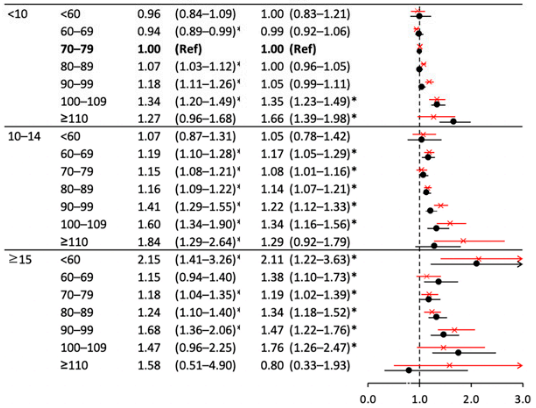

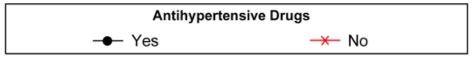

Figure S1. Hazard ratios for incidence of myocardial infarction, stroke, and death according to blood pressure, stratified by timed-up-and-go (TUG) test performance and the use of antihypertensive drugs.
